# Author Correction: COVID-19-related school closing aggravate obesity and glucose intolerance in pediatric patients with obesity

**DOI:** 10.1038/s41598-021-93375-6

**Published:** 2021-07-06

**Authors:** Eun Sil Kim, Yiyoung Kwon, Yon Ho Choe, Mi Jin Kim

**Affiliations:** Department of Pediatrics, Samsung Medical Center, Sungkyunkwan University School of Medicine, Seoul, Korea

Correction to: *Scientific Reports* 10.1038/s41598-021-84766-w, published online 09 March 2021

In the original version of this Article, Eun Sil Kim, Yiyoung Kwon, Yon Ho Choe and Mi Jin Kim were incorrectly affiliated with ‘Department of Medicine, Samsung Medical Center, Sungkyunkwan University School of Medicine, Seoul, Korea’. The correct affiliation is listed below.

Department of Pediatrics, Samsung Medical Center, Sungkyunkwan University School of Medicine, Seoul, Korea.

The original Article has been corrected.

